# Cerebral perfusion changes in acute subdural hematoma

**DOI:** 10.1007/s00701-023-05703-6

**Published:** 2023-07-18

**Authors:** J. Winkler, G. S. Piedade, C. Rubbert, B. B. Hofmann, M. A. Kamp, P. J. Slotty

**Affiliations:** 1 Department of Neurosurgery, Heinrich-Heine-University, Düsseldorf, Germany; 2 Department of Pediatric Neurosurgery, Nicklaus Children’s Hospital, University of Miami, 3100 SW 62nd Ave, Miami, FL 33155 USA; 3 Department of Diagnostic and Interventional Radiology, Heinrich-Heine-University, Düsseldorf, Germany; 4 Department of Neurosurgery, Friedrich-Schiller-University Jena, Jena, Germany

**Keywords:** Acute subdural hematoma, CT perfusion, Traumatic brain injury, Cerebral perfusion

## Abstract

**Introduction:**

Acute subdural hematoma (aSDH) is one of the main causes of high mortality and morbidity in traumatic brain injury. Prognosis is poor due to the rapid volume shift and mass effect. Cerebral perfusion is likely affected in this condition. This study quantifies perfusion changes in aSDH using early ER polytrauma CT with perfusion imaging (CTP).

**Methods:**

Data of 54 patients with traumatic aSDH were retrospectively collected. Glasgow Coma scale (GCS), perfusion parameters, therapeutic decisions and imaging data including hematoma thickness, midline shift, and hematoma localization were analyzed. The cortical perfusion parameters of each hemisphere, the area anterior to the hematoma (AAH), area below the hematoma (ABH), area posterior to the hematoma (PAH), and corresponding mirrored contralateral regions were determined.

**Results:**

We found a significant difference in Tmax in affected and unaffected whole-hemisphere data (mean 4.0 s vs. 3.3 s, *p* < 0.05) and a significantly different mean for Tmax in ABH and for the corresponding mirrored area (mABH) (mean 3.8 s vs. 3.1 s, *p* < 0.05). No significant perfusion changes in cerebral blood flow (CBF), cerebral blood volume (CBV), and mean transit time (MTT) were found.

**Conclusion:**

There was a significant elevation of time to maximum (Tmax) values in the underlying cortical area of aSDH. Possible pathophysiological explanations, the influence on immediate surgical decision-making and further therapeutic consequences have to be evaluated.

## Introduction

Traumatic brain injury (TBI) has a raw incidence of 10.1 cases per 100,000 persons for hospitalization [14]. Moderate and severe TBI are one of the main causes for death or lifelong severe impairment, especially in the age population from 29 to 45 [14]. Due to the high variability of trauma mechanisms, different patterns of brain injury are observed at admission in the emergency unit. The native CT scan performed at arrival at the emergency department gives information about acute bleedings, fractures, and volume shifts inside the skull. Many of the primary injury patterns are either directly or indirectly linked to changes in cerebral perfusion. Improving compromised perfusion is one of the main goals in neurological trauma surgery.

The main challenge in decision-making is to decide the overall necessity and urgency of surgical intervention of patients with TBI. Perfusion deficits are thought to play an important role in intracranial bleedings like aSDH, contusion hemorrhage or intracerebral hemorrhage due to the fast volume shifts with compression of brain tissue; these perfusion deficits are at least partly triggered by local or global intracerebral pressure (ICP) increase [10]. CTP assesses overall and local brain perfusion [9, 22]. CBF, CBV, MTT, and Tmax are the main parameters obtained and give information about tissue at risk, local blood flow, and general blood flow. There is a rising field of usability of the mentioned parameters in different perfusion related questions in neurosurgery, for example in chronic subdural hematoma or cranioplasty patients [19, 20], but the relevance for therapeutic decisions in patients with TBI is still unexplained.

There are few data describing perfusion deficits and how relevant these deficits are for therapeutic decisions. Because aSDH is the main determinant in short-time survival in TBI [12], we decide to focus this study on perfusion changes in patients with TBI and therefore aSDH as the leading pathology. For that prospectively collected early perfusion data of patients arriving at our emergency department with severe head trauma and aSDH are retrospectively analyzed.

## Methods

We collected data of patients with a traumatic aSDH as leading pathology and available CTP data presenting at our emergency department from 2013 to 2020. CTP is done in our institution as a standard of care for adult patients with suspected TBI and GCS < 8. Exclusion criteria were bilateral aSDH with the same size, imprecise CTP due to patient’s movements, and the absence of perfusion. We collected GCS values, perfusion parameters, laboratory results, therapeutic decisions and imaging data including hematoma width, midline shift, and hematoma localization. The study was approved by the Ethics Committee of the Medical Faculty of the Heinrich-Heine-Universität Düsseldorf (2020–1166).

CTP was performed on Somatom Definition AS 64 Slice CT-Scanner (Siemens Healthineers, Erlangen, Germany) with a saline chaser using 30 mL of contrast medium (Imeron 400, Bracco Imaging, Germany) bolus application with a 5 mL/s flow. Slices from the level of the orbital roof, the external meatus and cella media were selected to measure the perfusion. The perfusion data of the patients were imported to STROKETOOL-CT (Version 2.0, H.-J. Wittsack, http://www.digitalimagesolutions.de). We applied a motion correction to each data set. To estimate the arterial input function, which is the concentration of a contrast medium in an artery measured over time, we chose branches of the anterior and middle cerebral artery which had a clear time-intensity curve. The following calculation with the determined arterial input function of the perfusion parameters produced color maps for CBF, CBV, MTT, and Tmax.

The color maps were converted in numerical values in ECCETAngiotux CT 2 D (ECCET/AngioTux2D: Beck A., Aurich V institute for computer science/university of anatomy Düsseldorf). The regions of interest are marked with a circular border. The border has a width of 1 cm and runs with a distance of 2 mm parallel to the cortex to exclude the outer cerebrospinal fluid spaces. The superior sagittal sinus and the frontal part of the cerebral falx are also excluded. Within the border, the program computed an occipital clockwise rotation to analyze the perfusion parameters in 2-degree steps, which were exported to a table containing 180 regions of interest. It determined CBV, CBF, MTT, and Tmax for each region.

**Fig. 1:**
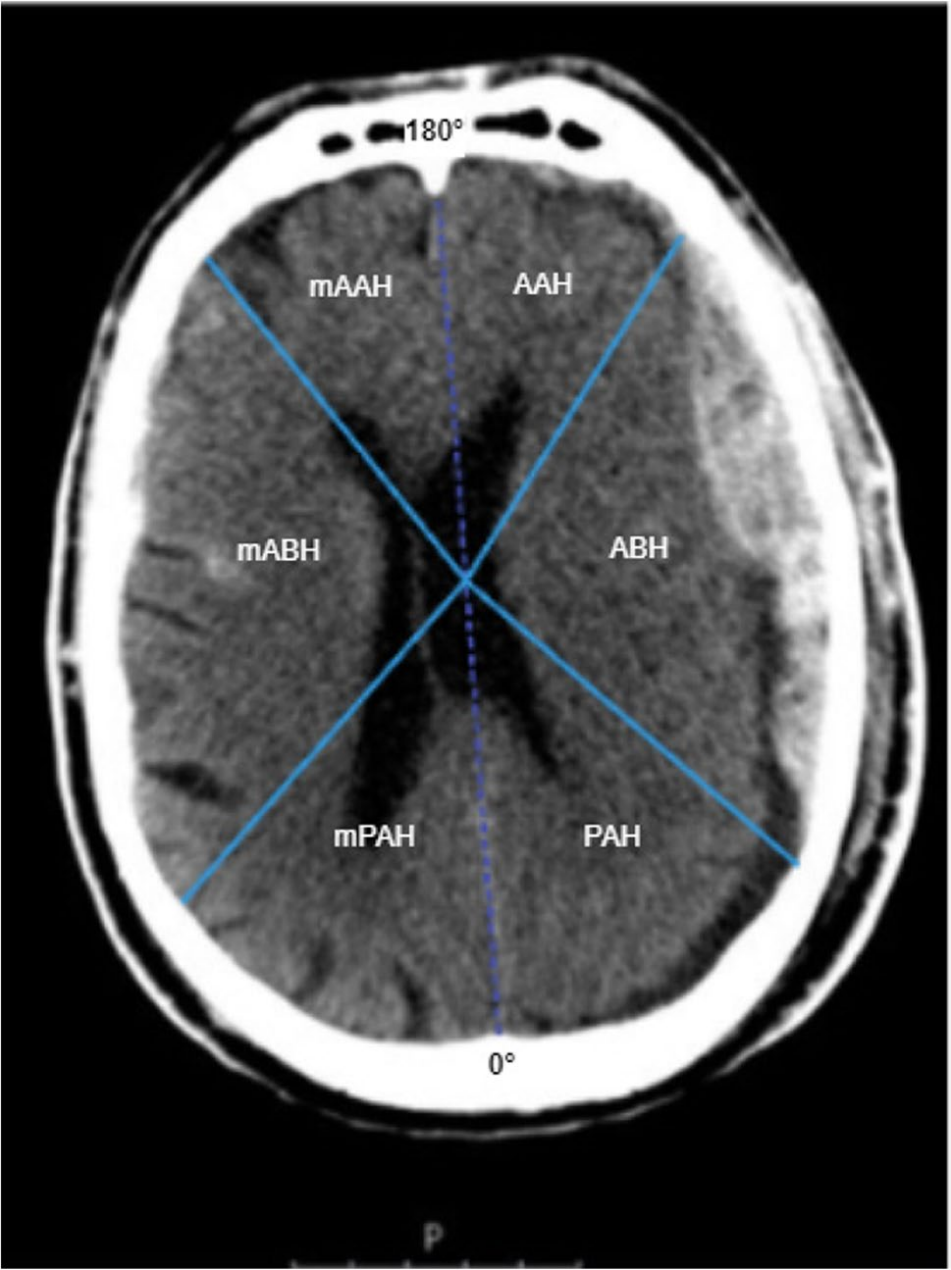
Native CT scan and identification of the analyzed areas

We used the Picture Archiving and Communication System of our institution and native CT images to analyze the angles of the ABH. We used the longitudinal cerebral fissure and frontal crista as a marker for 0-degree occipital and 180 degrees frontal and measured the angle of the ABH. We calculated the degrees for the AAH, PAH and the mirrored degrees (cf. Figure 1). With these values, the median for each parameter for the whole brain, for both hemispheres and for each selected region were calculated.

CBV describes the cerebral blood volume in ml/g and CBF is commonly measured in ml/100 g/min. MTT is defined as $\frac{CBV}{CBF}$ and is measured in seconds. Tmax is also measured in seconds and describes the time span between the arterial input function and the arrival in brain parenchyma. It can be computed by deconvoluting the arterial input function [5].

### Statistics

Statistical analysis was performed in Python (Version 3.9.10). To compare the cerebral perfusion in different areas a two samples independent *t*-test was used. The significance level was set to $\alpha =0.05$. The analysis of correlations was performed with the Pearson correlation coefficient.

## Results

We collected data of 54 patients with aSDH as leading pathology, their ages ranged from 17 to 92 years and the median age was 60.5 years (Table 1). Aside from aSDH, 31 patients suffered additionally from traumatic subarachnoid hemorrhage, eleven patients showed a contusion hemorrhage, and six patients had an intracerebral hemorrhage. Other concurrent cranial injury patterns were epidural hematoma and fractures of the skull. Forty-six patients had a GCS < 8 and arrived intubated in the emergency unit. Nine patients had an elevated INR due to oral anticoagulation. The main trauma mechanism was falling downstairs (11 patients). Other trauma mechanisms were falls from height, traffic accidents, and accidents with sharp tools.

**Table 1:** Patient population by treatment concept

	Operated	Conservative	Palliative	All
*n*	28	20	6	54
Median age (in years)	59	48.5	66.5	60.5
Median GCS	3	5	5	4
Median hematoma thickness (in mm)	11.15	4.25	9.0	7.6
Median midline shift (in mm)	5.5	0	11.5	3.0
Median INR	1.15	1.10	1.20	1.20

**Fig. 2:**
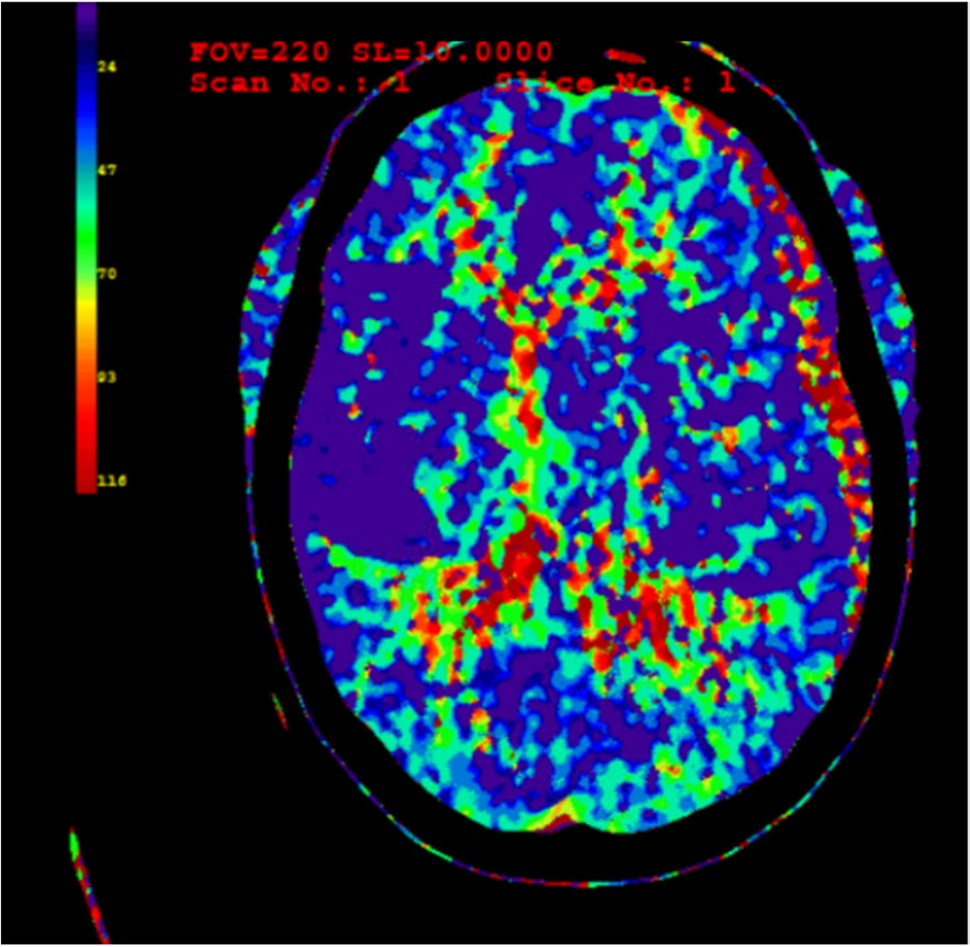
CTP of a patient with left hemispherical aSDH

**Fig. 3:**
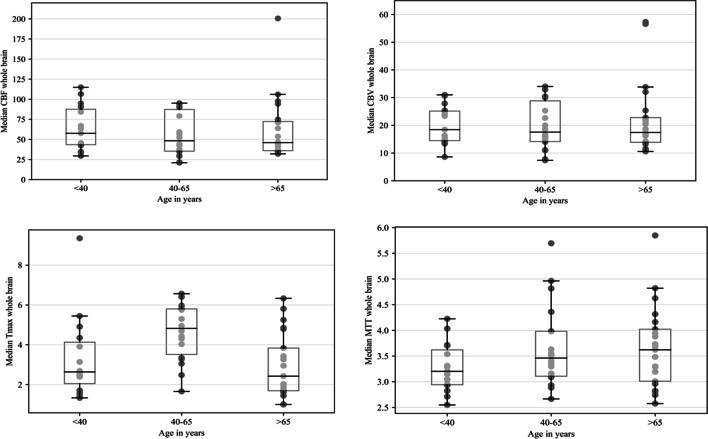
Boxplots of the median CBF, CBV, Tmax, and MTT perfusion values measured in the whole brain and differentiated by age. In the middle aged group (40–65 years) exists an additional outlier value at CBF = 352.7 and CBV = 114.8. Both values are not included in the plots due to illustration purposes

There was a significant difference in Tmax in affected and unaffected whole-hemisphere data (mean 4.0 s vs. 3.3 s, *p* < 0.05). There was also a significantly different mean for Tmax in ABH and for the corresponding mABH (mean 3.8 s vs. 3.1 s, *p* < 0.05) (Table 2). This difference was present for the whole study population and mainly seen in the age group of 40–65 years. An elevated Tmax in ABH in CTP imaging is illustrated in Fig. 2. The Tmax elevation can be visually detected by having the same shape in CTP as the aSDH in native CT scan. No significant perfusion changes in cerebral blood flow (CBF), cerebral blood volume (CBV), and MTT were found (mean CBF 69.7 ml/100 g/s vs. 68.8 ml/100 g/s, *p* = 0.927; CBV 2.31 ml/100 g vs. 2.29 ml/100 g, *p* = 0.916; MTT 3.5 s vs. 3.5, *p* = 0.879). The median values for the whole brain were CBF = 46.1 ml/100 g/s, median CBV = 1.8 ml/100 g, median MTT = 3.38 s, and median Tmax = 4.61 s (Fig. 3). Tmax in the AAH was significantly higher than in the mirrored area for the whole study population (AAH 43.96 s vs. mAAH 34.54 s, *p* < 0.05). *t*-test with the PAH and the mirrored area showed no significance. When different lesion patterns were compared — patients with pure aSDH vs. patients with aSDH and other traumatic lesions — no significant difference was found.

**Table 2:** *t*-test of cerebral perfusion mean values in ABH and mABH grouped by age and treatment

		CBF (ml/100 g/min)			CBV (ml/g)			Tmax (in seconds)			MTT (in seconds)		
		ABH	m-ABH	*p*-value	ABH	m-ABH	*p*-value	ABH	m-ABH	*p*-value	ABH	m-ABH	*p*-value
< 40 years (*n* = 15)	Operated (*n* = 6)	71.0	63.82	0.779	22.18	20.16	0.75	4.67	3.85	0.706	3.13	3.34	0.518
	Conservative (*n* = 9)	72.51	73.87	0.925	22.01	21.65	0.92	2.16	2.05	0.773	3.30	3.12	0.513
	Palliative (*n* = 0)	-	-	-	-	-	-	-	-	-	-	-	-
	All	71.9	69.85	0.872	22.08	21.06	0.745	3.12	2.77	0.675	3.24	3.23	0.989
40–65 years (*n* = 18)	Operated (*n* = 10)	81.41	86.69	0.857	27.55	28.83	0.891	4.99	3.35	0.04*	3.57	3.61	0.92
	Conservative (*n* = 5)	57.27	54.77	0.841	21.18	19.04	0.631	4.32	4.14	0.798	3.54	3.61	0.911
	Palliative (*n* = 3)	39.04	47.54	0.384	15.11	18.83	0.435	7.46	4.41	0.033*	4.26	4.28	0.975
	All	67.64	71.3	0.832	23.71	24.45	0.893	5.21	3.75	0.01*	3.68	3.72	0.877
> 65 years (*n* = 21)	Operated (*n* = 12)	53.45	58.72	0.655	19.85	20.45	0.878	4.00	3.07	0.294	3.84	3.57	0.332
	Conservative (*n* = 6)	101.68	81.63	0.72	26.42	24.36	0.861	1.69	1.79	0.819	3.14	3.26	0.685
	Palliative (*n* = 3)	71.81	63.89	0.767	31.41	28.81	0.897	2.98	2.95	0.953	4.14	4.32	0.894
	All	69.85	66.0	0.822	23.38	22.76	0.893	3.19	2.69	0.376	3.69	3.59	0.708
All		69.69	68.84	0.927	23.13	22.85	0.916	3.85	3.06	0.046*	3.56	3.53	0.879

*CBF* cerebral blood flow, *CBV* cerebral blood volume, *Tmax* time to maximum, *MTT* mean transit time, *ABH* area below hematoma, *mABH* mirrored area below hematoma. Significant results are marked with a *. Bold indicates *p *< 0.05

There is a moderate but significant correlation between the maximal width of aSDH and Tmax in ABH (*r* = 0.35, *p* = 0.0092) and between the level of midline shift and Tmax in ABH (*r* = 0.41,* p* = 0.0019). There is no correlation between INR and Tmax in ABH (*r* = 0.05, *p* = 0.7188), nor between GCS and Tmax in ABH (*r* =  − 0.23, *p* = 0.1196).

In the examined group, 28 patients were operated, 20 patients were managed conservatively, and 6 patients got a palliative treatment (Table 1). Surgical procedure for hematoma evacuation was either a decompressive craniectomy or, in the case of good primary neurological status, a craniotomy. Patients who underwent surgery had a lower median GCS value (median GCS = 3) than patients who were treated conservatively (median GCS = 5) or palliatively (GCS = 5). The median age of the conservatively managed patients was younger (48.5 years) than the surgically managed patients (59 years), who were younger than the palliatively managed patients (66.5 years). The median thickness of the hematoma was 11.15 mm in the surgery group and 4.25 mm in the conservative group. Conservatively treated patients had a median midline shift of 0 mm, while operated patients had a shift of 5.5 mm. INR was similar in each group (operated 1.15, conservative 1.10, palliative 1.20). In the affected hemisphere, Tmax was significantly higher in patients treated surgically than in patients managed conservatively, whereas the surgical decision was never based on perfusion results (mean Tmax affected hemisphere surgery 4.7 s vs. mean Tmax affected hemisphere conservative 2.7 s, *p* < 0.001). In addition, we compared Tmax in the affected hemisphere in patients who survived and patients who did not. A difference of the mean values in Tmax (mean Tmax affected hemisphere survival 3.5 s vs. mean Tmax affected hemisphere clinical death 5.2 s, *p* < 0.05) was detected. The mean Tmax of patients who were treated with palliative care was 5.2 s.

## Discussion

CTP is well-known mainly in the setting of acute ischemic stroke and detects the penumbra, which is the tissue in the neighborhood of the acute insult and may be saved by early mechanical or drug-related recanalization therapy [4]. In the DEFUSE-2 study, an elevated Tmax predicted the infarct growth and was a predictive marker for poor outcome, even if the arteries became recanalized [11]. Recent articles have shown promising results using perfusion imaging to diagnose tissue at risk also in the case of traumatic brain injury [2, 23].

In native CT scan, the tissue at risk is hard to delineate and cerebral hematomas like contusions might not be observed at day one [23]. By delineating the tissue at risk in CTP, which is mostly indicated by increased Tmax, early and objective data may possibly contribute to surgical decisions when correlated with clinical aspects. In the setting of severe TBI, different articles point out that not only the primary insult leads to brain damage, but also a secondary ischemic injury related to cerebral edema and local intracranial hypertension [7, 23]. Bendinelli et al. described brain ischemia related to TBI, which is underdiagnosed, but important for decision-making. In his study, CTP data changed the management of three TBI patients with massive brain ischemia that showed only minimal changes in native CT scan [2]. In the present study, operated patients had a higher Tmax and patients selected for palliative care had an extremely elevated Tmax.

Radiologists already use Tmax as an indicator for tissue at risk. Wintermark et al. showed that the detection of tissue at risk is an important prognostic indicator [23]. They included 130 patients with severe TBI and evaluated them with the Glasgow Outcome scale 3 months after injury. The authors found a correlation between higher MTT and a worse outcome and a positive correlation between higher number of arterial territories with high CBV and a better outcome. Furthermore, 18 patients of the study population suffered from aSDH and showed elevated MTT values and lower CBF and CBV values when compared to the control group [23]. In our study, no significant difference in CBV, CBF and MTT was detected, but a higher Tmax was present and correlated to clinical death. No data about long-term outcome are available, but extremely elevated Tmax values ≥ 65 s could only be measured in patients with GCS ≤ 4 and expected very poor outcome. Further, Wintermark et al. compared the perfusion values in different bleedings. The strongest reduction of perfusion was found in patients with an epidural hematoma [23]. Also, intracranial hypertension and cerebral contusions lead to decreased perfusion values. Many studies describe changes in perfusion with CBV, CBF, and MTT in different bleedings, but Tmax is not mentioned [7, 8, 23]. Because of the retrospective design of the current study and the consequential small data set, it was not possible to choose patients with only aSDH. It remains unclear how the mentioned co-pathologies influence the value of Tmax.

Because Tmax is a complex marker and its clinical relevance is still unknown, there is little evidence about its role in TBI. Calamante et al. described Tmax as the arrival delay between the arterial input function and the tissue contrast agent [5]. To calculate Tmax, the arterial input function must be deconvoluted. It is necessary to combine the deconvolution with a regularization to smooth the function. This regularization is influenced by MTT, CBV and the signal-to-noise ratio. For this reason, the deconvoluting algorithm depends on the specific implementation in each institution [5, 21]. In addition to that, CBF, CBV, and MTT are mathematical constructs which are not directly measured and depend on algorithms [21]. Therefore, no generalized norm-values in CTP can be estimated [5, 21], and it is difficult to compare these values as quantitative measurements. For example, CBV may be different in the same patient, because the amount of brain volume in one voxel can differ. In comparison to the other values, Tmax is a time delay-dependent factor detecting a delay of the arrival of contrast bolus in brain tissue. Therefore, it provides relevant additional information about the patient’s hemodynamical status [5]. Several studies mention decreased CBF and CBV and an increased MTT in patients with TBI and SDH [7, 23]. In our study, no significant difference in CBV, CBF and MTT could be detected in the compared brain areas. It is unclear how aSDH affects the brain tissue; therefore, no “healthy” tissue could be defined. Because no normal perfusion values exist, it was not possible to diagnose an elevation or reduction of perfusion values in the whole brain.

The cohort was divided into three groups. As each patient has a different incidence of aSDH, it is not meaningful to compare mean Tmax between groups, but the middle-aged group showed a significant increase in Tmax ABH. Biagi et al. compared cerebral perfusion in three age-cohorts (children, teenagers, and adults) with CBF value measured by the arterial spin labeling technique in magnetic resonance imaging [3]. They describe a reduction of CBF especially in the grey matter with increasing age [3]. Most people in the elderly suffer from diseases such as hypertension [15], which leads to confluent white matter lesions [24]. Bastos-Leite et al. showed that these lesions are associated with a reduced brain perfusion [1]. This underlines the importance of considering that perfusion values differ between age groups and that even minor injuries in older people may lead to a worse outcome than more severe injuries in younger patients [16].

We explain the difference of Tmax between ABH and mABH with a nonfunctional autoregulation. Slotty et al. showed that autoregulation functions in chronic SDH probably because the hematoma grows slowly. They found an elevated CBV and CBF in ABH and no difference of Tmax and MTT between ABH and mABH [20]. This leads to the possible explanation that CBV and CBF do not adapt quickly in the case of aSDH because of its sudden growth and therefore Tmax elevates. This could reveal the absence of autoregulation with the consequence of hypoperfused brain tissue. It remains unclear whether the autoregulation defect is based on a higher ICP due to mass effect or on the trauma and following biochemical pathways. Today, therapeutic decisions are based in part on the detection of mass shift on native CT scans. Table 1 shows that patients with a large hematoma thickness or midline shift were more likely to be treated surgically. Salvant et al. mentioned the importance of mass effect and elevated ICP in aSDH. They compared two groups of patients: group one had major lesions with mass effect or midline shift after TBI and group two consisted of TBI patients without mass effect [17]. Group one with mass effect showed comparatively a decreased CBF in the first 48 h of injury. The patients with aSDH often developed secondary brain ischemia, measured with the cerebral metabolic rate of oxygen consumption. As mentioned before, Tmax is a delay-dependent value, so its elevation in ABH could be based on an elevated ICP especially in the affected region. Unfortunately, ICP values were not measured in our study.

The expected elevation of ICP in aSDH was observed in some studies and was often caused by brain swelling [18]. De Souza et al. mentioned that brain swelling results in a disproportion between hematoma thickness and midline shift [6]. This study included 114 patients with aSDH. De Souza et al. analyzed the Zumkeller index ($ZI=midline\;shift-hematoma\;thickness$), an indicator of brain swelling, as a prognostic factor and found out that a ZI > 3 is a predictor of mortality in patients with aSDH with odds ratio of 8.12. Nearly half of the patients with a ZI > 3 also had an intraventricular hemorrhage, which is associated with a poor prognosis [6, 13]. This supports the correlation between midline shift and Tmax ABH found in our study.

### Limitations

As limitations of the study we identify the retrospective design and the fact that no long-term data are available. Although very difficult in such a study, ICP measurements would have been of great value. Another limitation is that it was not possible to calculate the impact of underlying pathologies like contusion hemorrhage, intracerebral bleedings, etc. on Tmax. For that, we plan to conduct further studies.

## Conclusions

Tmax seems to be a relevant marker for the outcome after aSDH, but its therapeutical importance remains unclear. Further prospective studies are needed with a larger and more selective study population. Also, studies which calculate the impact of other intracranial bleedings like contusion hemorrhage or intracerebral bleedings are needed and scheduled. In the future, Tmax may possibly become an influencing factor for surgical management decisions when clinical aspects are not clear.

## Data Availability

Image data were extracted from the PACS of the Düsseldorf University Hospital. All clinical data were obtained from Medico of the Düsseldorf University Hospital.

## Abbreviations

aSDH: Acute subdural hematoma
CTP: Computer tomography with perfusion imaging
GCS: Glasgow Coma scale
AAH: Area anterior to the hematoma
ABH: Area below hematoma
PAH: Area posterior to the hematoma
mAAH: Mirrored area anterior the hematoma
mABH: Mirrored area below the hematoma
mPAH: Mirrored area posterior the hematoma
CBF: Cerebral blood flow
CBV: Cerebral blood volume
MTT: Mean transit time
Tmax: Time to maximum
TBI: Traumatic brain injury
ICP: Intracerebral pressure
